# Clarithromycin and Pantoprazole Gastro-Retentive Floating Bilayer Tablet for the Treatment of Helicobacter Pylori: Formulation and Characterization

**DOI:** 10.3390/gels9010043

**Published:** 2023-01-04

**Authors:** Ghufran Ullah, Asif Nawaz, Muhammad Shahid Latif, Kifayat Ullah Shah, Saeed Ahmad, Fatima Javed, Mulham Alfatama, Siti Aisyah Abd Ghafar, Vuanghao Lim

**Affiliations:** 1 Advanced Drug Delivery Laboratory, Gomal Centre of Pharmaceutical Sciences, Faculty of Pharmacy, Gomal University, Dera Ismail Khan 29050, Pakistan; 2 Institute of Biotechnology and Microbiology, Bacha Khan University, Charsadda 24420, Pakistan; 3 Department of Chemistry, Shaheed Benazir Bhutto Women University, Charsadda Road Larama, Peshawer 25000, Pakistan; 4 Faculty of Pharmacy, Universiti Sultan Zainal Abidin, Besut Campus, Besut, Terengganu 22200, Malaysia; 5 Department of Basic Science, Faculty of Dentistry, Universiti Sains Islam Malaysia, Tower B, Persiaran MPAJ, Jalan Pandan Utama, Pandan Indah, Ampang, Kuala Lumpur 55100, Malaysia; 6 Advanced Medical and Dental Institute, Universiti Sains Malaysia, Bertam, Kepala Batas, Penang 13200, Malaysia

**Keywords:** pantoprazole, clarithromycin, immediate-release layer, controlled-release floating layer, floating drug delivery

## Abstract

Bilayer/multilayer tablets have been introduced to formulate incompatible components for compound preparations, but they are now more commonly used to tailor drug release. This research aimed to formulate a novel gastro-retentive tablet to deliver a combination of a fixed dose of two drugs to eliminate *Helicobacter pylori* (*H. pylori*) in the gastrointestinal tract. The bilayer tablets were prepared by means of the direct compression technique. The controlled-release bilayer tablets were prepared using various hydrophilic swellable polymers (sodium alginate, chitosan, and HPMC-K15M) alone and in combination to investigate the percent of swelling behavior and average drug release. The weight of the controlled-release floating layer was 500 mg, whereas the weight of the floating tablets of pantoprazole was 100 mg. To develop the most-effective formulation, the effects of the experimental components on the floating lag time, the total floating time, T 50%, and the amount of drug release were investigated. The drugs’ and excipients’ compatibilities were evaluated using ATR-FTIR and DSC. Pre-compression and post-compression testing were carried out for the prepared tablets, and they were subjected to in vitro characterization studies. The pantoprazole layer of the prepared tablet demonstrated drug release (95%) in 2 h, whereas clarithromycin demonstrated sustained drug release (83%) for up to 24 h (F7). The present study concluded that the combination of sodium alginate, chitosan, and HPMC polymers (1:1:1) resulted in a gastro-retentive and controlled-release drug delivery system of the drug combination. Thus, the formulation of the floating bilayer tablets successfully resulted in a biphasic drug release. Moreover, the formulation (F7) offered the combination of two drugs in a single-tablet formulation containing various polymers (sodium alginate, chitosan, and HPMC polymers) as the best treatment option for local infections such as gastric ulcers.

## 1. Introduction

In oral drug delivery systems, sustained-release (SR) dosing forms are essential for enhancing patient compliance, lowering side effects, and boosting pharmaceutical bioavailability. When it comes to drugs having a small window of absorption in the upper GI tract, the short and erratic stomach emptying interval may impede SR systems from delivering a full release of the drug to its absorption site. To address this disadvantage, gastro-retentive (GR) technologies have been introduced. The absorption of drugs in the proximal GI tract is improved by GR delivery systems because they can remain in the stomach for prolonged periods compared to the conventional dosage forms [1]. GR systems can be divided into expandable, floating, mucoadhesive, and high-density systems based on the gastric retention strategy. In effervescent floating systems, it is feasible to generate floatation by creating gas and trapping air inside the polymer matrix; however, there may be a delay before the system floats [2].

A gastro-retentive drug system with a continuous residence in the stomach is essential for drug molecules that function in the stomach, possess an absorption window in the upper GIT, and have a degradation tendency in the intestinal or colonic surrounding [3]. This method can also be used to progressively bring pharmaceuticals into the small intestine window, lowering dosing frequencies by extending drug activities, weakening drug of the inactivation in the lower GI tract, and reducing the drug’s effects on the flora of the lower intestine [4]. A floating drug delivery approach is the most promising among the gastro-retentive dose forms because it does not interfere with the GIT’s motility, and several of these floating dosage forms are available on the market [5,6]. A biphasic delivery method uses a composite bilayer tablet with two separate release layers in an effort to give medication at various rates or concurrently release two drugs. This has several advantages, including the ability to combine two poorly compatible pharmaceuticals into one system, the ability to release two drugs with appropriate release profiles at the same time, the ability to boost therapeutic efficacy through a synergistic effect, the ability to reduce the dosing unit burden, and the ability to improve patient compliance [7].

Multilayer tablets have recently been proposed to improve the compatibility of different compound characteristics in a single dose, hence ameliorating the efficacy and patient compliance by reducing the dose load [8]. Furthermore, the layered tablet technique can modulate the release profiles [9]. One layer, for example, is designed for immediate release, while the others are for prolonged release. The instant-release layer is designed to disintegrate rapidly and transiently post-oral administration, delivering enough drug concentration for immediate action. In contrast, the controlled-release layer is disintegrated slowly in the gastrointestinal tract to preserve a constant blood level. Polymers have played an important role in designing controlled drug delivery systems. Polymer-based delivery provides safe passage for pharmaceuticals and delivers the drug at the target site. Polymers such as chitosan, alginate, and HPMC gain more importance in drug delivery due to their biodegradability and biocompatibility. In the current study, these polymers were used alone and in combination to design the bilayer floating tablets of clarithromycin and pantoprazole.

*Helicobacter pylori* (*H. pylori*) are Gram-negative bacteria that grow in microaerophilic environments and have 4–6 polar flagella. They are found abundantly on the mucosal surface of the stomach and are able to penetrate the mucosal layer to continue developing. *H. pylori* infection is frequent in young people in underdeveloped nations, with a global prevalence of more than 50% [10]. In developed countries, *H. pylori* are linked to gastritis (which can progress to stomach ulcers and gastric cancer), malnutrition, chronic diarrhea, and underlying illnesses such as enteric infections (cholera and typhoid fever) [11]. To treat *H. pylori* infections, antibacterials such as amoxicillin, metronidazole, tetracycline, and clarithromycin are routinely utilized with bismuth salts and proton pump inhibitors. However, side effects and antibiotic resistance cause several individuals to fail to respond to treatment [12].

Clarithromycin is a macrolide antibacterial that causes the inhibition of protein synthesis by binding to the 50s sub-units of the ribosomal microorganism. Clarithromycin is a drug of choice for treating ulcers and lower and upper GIT bacterial infections. Due to its stability in acid pH and efficient absorption in the stomach, this antibiotic is an alternative for eliminating *H. pylori* compared to other macrolides [13]. A sustained drug delivery system is suitable for achieving the maximum amount of drug release in a slow manner while minimizing the fluctuation of the drug concentration in the plasma. Clarithromycin has a relatively long clearance half-life (3.3 to 4.9 h), which is appropriate for developing a sustained drug delivery formulation [14].

Pantoprazole, a proton pump inhibitor (PPI), is responsible for inhibiting the potassium/hydrogen ions of the ATPase enzyme at the gastric parietal cell secretory surfaces. This effect inhibits the baseline and provokes stomach acid secretion, regardless of the stimulus. Pantoprazole’s antisecretory impact persists longer than 24 h due to its irreversible binding to the (H+, K+)-ATPase enzyme, which requires new enzymes to be produced to continue acid secretion. The recommended dose of pantoprazole for treating GERD, gastric ulcer, and duodenal ulcer is 45 mg per oral route and is prescribed for 8 to 12 weeks. It is desirable for floating drug delivery systems because it acts locally and has a half-life of 1 to 2 h. These properties of clarithromycin and pantoprazole are considered suitable candidates for gastro-retentive floating drug delivery systems. Previously, Jain et al. prepared and evaluated bilayer tablets of pantoprazole and clarithromycin using various grades of HPMC [15]

The present study aimed to investigate the feasibility of designing and applying a bilayer tablet strategy as an immediate- and extended-release formulation of pantoprazole and clarithromycin, respectively. This study used natural, biodegradable, and biocompatible polymers, chitosan, alginate, and HPMC, to control the swelling, floating, and in vitro release of the drugs. The formulation and preparation were conducted, and the in vitro release of the prepared bilayer floating tablets were examined, followed by release kinetics studies.

## 2. Results and Discussion

### 2.1. Drugs’ and Excipients’ Compatibility

**Figure 1:**
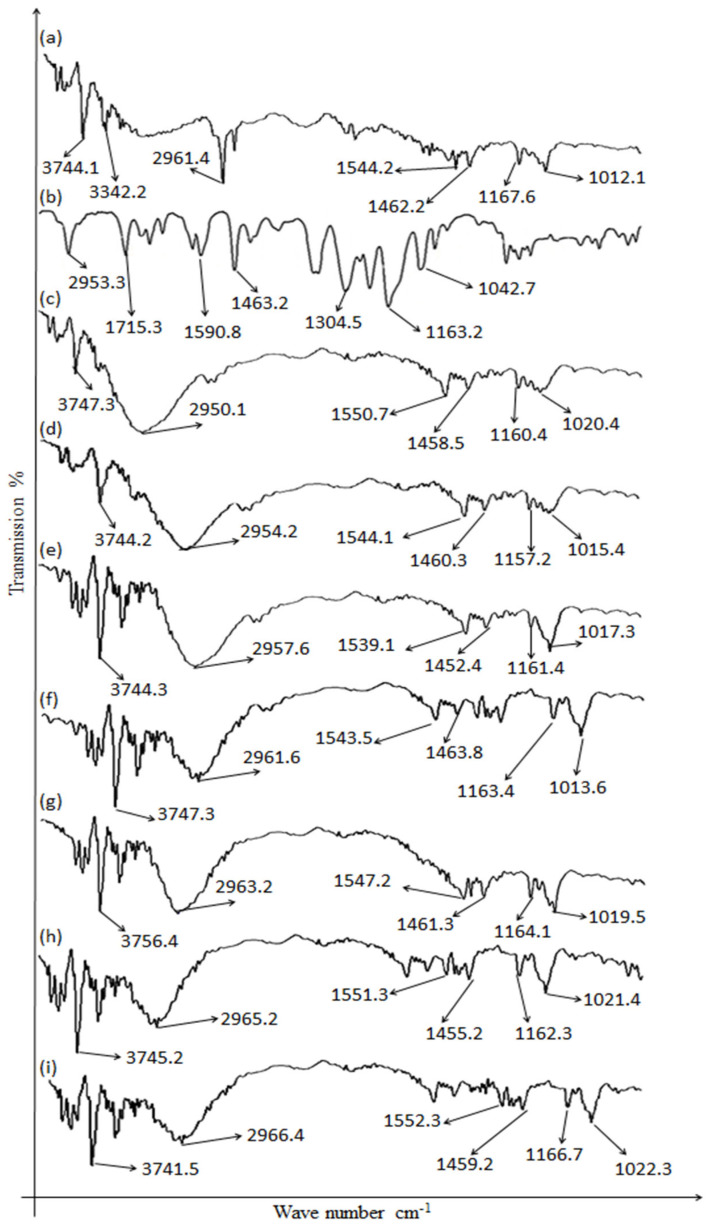
FTIR spectra of (**a**) clarithromycin, (**b**) pantoprazole sodium, (**c**) F1, (**d**) F2, (**e**) F3, (**f**) F4, (**g**) F5, (**h**) F6, and (**i**) F7.

The ATR-FTIR and DSC studies were conducted to investigate the probable interactions between the drugs and excipients used in the bilayer tablet formulations (F1–F7) (Figure 1). The pantoprazole characteristic peaks were found at 1715.3 N-H bending vibration, 1590.8 (C-N vibrations) aromatic primary, 1463.2 sulfur compound (sulfonyl chloride), and 1042.7 (O-H bending and C-O stretching vibration primary alcohols) [16], whereas the characteristic peaks of clarithromycin were found at 3744.1 cm^−1^ alcohol O-H stretching, 2961.4 cm^−1^ carboxylic acid O-H stretching, 1544.2 cm^−1^ nitro compound N-O stretching, 1462.2 alkane C-H bending, and 1012.1 cm^−1^ primary alcohol C-O stretching [17]. The characteristic peaks of clarithromycin and pantoprazole were found in all the formulation combinations with a slight shift in wavenumber, showing no chemical incompatibility. The results showed that the formulation of the bilayer tablets of pantoprazole and clarithromycin (F1–F7) had all the characteristic peaks of pantoprazole and clarithromycin and the characteristic peaks were preserved, indicating that the formulations were safe for use and no physical/chemical incompatibilities were observed. It was found that the drugs, pantoprazole and clarithromycin, and the excipients used in the preparation of the bilayer tablets were compatible and safe for use.

**Figure 2:**
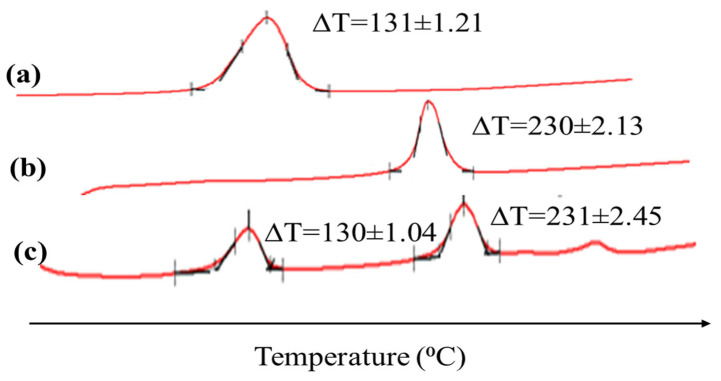
DSC thermograms of (**a**) pantoprazole, (**b**) clarithromycin, and (**c**) formulation F7.

The DSC thermograms of the pure drugs (pantoprazole and clarithromycin) along with the optimized formulation (F7) are shown in Figure 2. An endothermic peak at 131 °C was observed for pure pantoprazole and 230 °C for clarithromycin, corresponding to their melting points. There was no significant difference in the thermograms of the pure drugs and formulations, suggesting that there is no interaction between the drugs and excipients used in the formulation of the tablets.

### 2.2. Flow Behavior

The flow behavior of powders is of utmost importance in the formulation of tablets. The flow behavior has a role in the elegance and smooth finishing of the final product. The ideal finished product exhibited a good flow behavior of the powder. The ideal flow properties of the powder were investigated by subjecting the formulation of the tablets to pre-compression studies. The value of Hausner’s ratio, Carr’s index, tapped density, bulk density, and the angle of repose were evaluated for the formulated tablets. The results of the floating tablets’ immediate- and sustained-release layers are shown in Table 1 and Table 2. All three formulations of the immediate-release layer of pantoprazole were analyzed. The compressibility index value ranged between 6.944% and 8.298%; Hausner’s ratio ranged between 1.075 and 1.09; the angle of repose ranged from 29.47 θ to 29.70 θ (Table 1). The flow parameters were also observed for a slow-release floating layer of clarithromycin HCl. The compressibility index values ranged between 8.432% and 14.80%; Hausner’s ratio ranged between 1.092 and 1.174; the angle of repose ranged from 29.4 θ to 30.5 θ (Table 2). All the formulated tablets exhibited values within the acceptable limits of the United States Pharmacopeia [18]. It was shown that all the formulations had good flow behavior and lied within the specified acceptable limits per the USP guidelines, 2019, which is important for tablet preparations.

### 2.3. Post-Compression Studies

The floating tablets were prepared by a single-punch tableting machine. In the case of the immediate-release layer of pantoprazole, the formulation F2-IR was selected as an optimized formulation compared to the other formulated tablets. The criteria for the selection of the IR layer were the in vitro disintegration period and in vitro drug delivery. The fabricated immediate layer was further processed with a controlled layer of clarithromycin. The prepared bilayer tablets were subjected to the following physical and chemical tests.

#### 2.3.1. Hardness

The hardness of the optimized bilayer tablets was evaluated by a hardness tester (Erweka TB 24 Apparatus, Germany). The hardness value of the optimized formulated tablets ranged between 67 and 71 N (Table 2). These values were in an acceptable range and exhibited enough strength to withstand any breakage during handling, transportation, and storage [18].

#### 2.3.2. Uniformity of Weight

An analytical balance was used for the measurement of the weight uniformity of the formulated tablets. Randomly, 10 tablets were selected from the batch, and the weight was measured and averaged. The value of the weight uniformity for the optimized tablets ranged between 598 and 605 mg (Table 2). The values were very close to each other, indicating that the prepared tablets showed weight uniformity.

### 2.4. Uniformity of Drug Content

UV–visible spectroscopy was used for the determination of the content uniformity of pantoprazole, whereas the HPLC method was used for clarithromycin HCl. The drug content value of pantoprazole ranged from 97.3–100.5%, whereas the drug content value of clarithromycin ranged from 97.9–100.7% (Table 2). The formulated tablets showed results within an acceptable range (BP standards). The official limits of the drug contents approved by the BP standards range between 85 and 115% (BP Pharmacopeia, 2009) [19,20].

#### 2.4.1. Dimensions

The diameter of the formulated optimized bilayer tablets was evaluated using a vernier caliper (Table 2). Dies of 15 mm diameter were used for the formulation of the bilayer floating tablets. The diameter of the formulated tablets was 15 mm. The thickness of the formulated tablets was also evaluated using a vernier caliper. The thickness of the formulated tablets ranged between 2.65 and 2.75 mm. The closeness of the values of the formulated tablets showed a uniform thickness.

#### 2.4.2. Friability

A friability test was performed on all prepared formulated tablets. The formulated tablets showed that the results lied within the limits (Table 2). The friability test of the formulated tablets was less than 1%, which is in accordance with the value of the official BP, 2020. These results indicate that the prepared tablets exhibited no splitting, breaking, or cracking, which ensured that the prepared tablets were strong enough to withstand wear and tear [21].

#### 2.4.3. Tablet Floating Capacity

Gastro-retentive floating tablets were prepared to float over the surface of the gastric medium for an extended period of time without altering the gastric emptying time. In a floating drug delivery system, early stomach emptying of the dose form can be minimized. The density of the floating tablets must be less than the density of the gastric contents (1.004 g/mL) [22]. This can be achieved by enhancing the total time of floating and minimizing the lag time for floating.

A 0.1 N HCl solution was used to assess the floating lag time and overall floating time of the prepared tablets. The floating lag time of the formulated tablets ranged from 12 s (F4) to 1 min and 38 s (F6). The total floating time of the formulated tablets ranged from 15 h (F5) to 24 h (Table 3). The results indicate that all prepared tablets showed a minimum floating lag time and retained good floating effects.

### 2.5. Swelling and Erosion Studies

**Figure 3:**
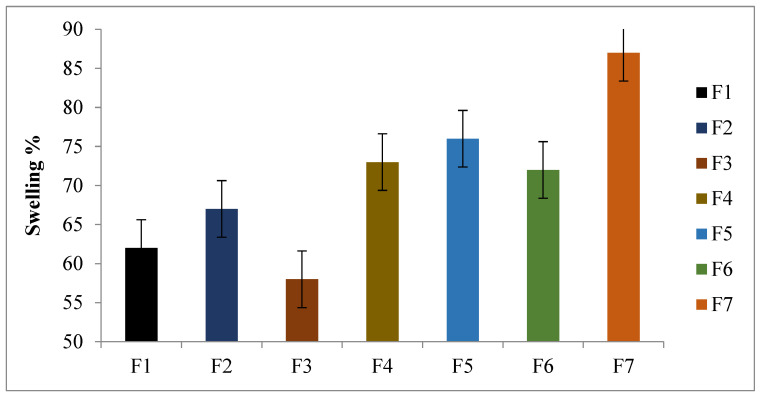
Percent swelling of formulations (F1–F7). Data are expressed as the mean ± SD; *n* = 3, one-way ANOVA followed by the post hoc Tukey test (*p* < 0.05), F1 vs. F7 (*p* < 0.05).

The swelling and erosion profiles of polymers such as HPMC-, chitosan-, and alginate-based floating tablets were used to construct a correlation with the drug release attributes and release mechanisms. The density of the formulated floating tablets was low compared to the density of the gastric fluid in the stomach (1.004 g/cm^3^). The HCl solution (0.1 N) was used for the evaluation of the swelling test. The formulated tablets were immersed in gastric fluids, travelled to the surface of the fluid medium, and swelled upon hydration by using gel-forming polymers. The prepared formulations exhibited a percent swelling value ranging from 58% to 87% (Figure 3). The addition of sodium alginate, chitosan, and HPMC in formulation F7 showed increased water uptake and, hence, a higher swelling index. The maximum swelling index of 87% was exhibited by formulation F7, containing sodium alginate, chitosan, and HPMC in the same ratio of 1:1:1. A thick gel-like barrier layer was formed with the maximum percent swelling that retarded and increased the diffusion path length for the drug molecules. The formation of a viscous gel layer further retarded the drug release [23].

**Figure 4:**
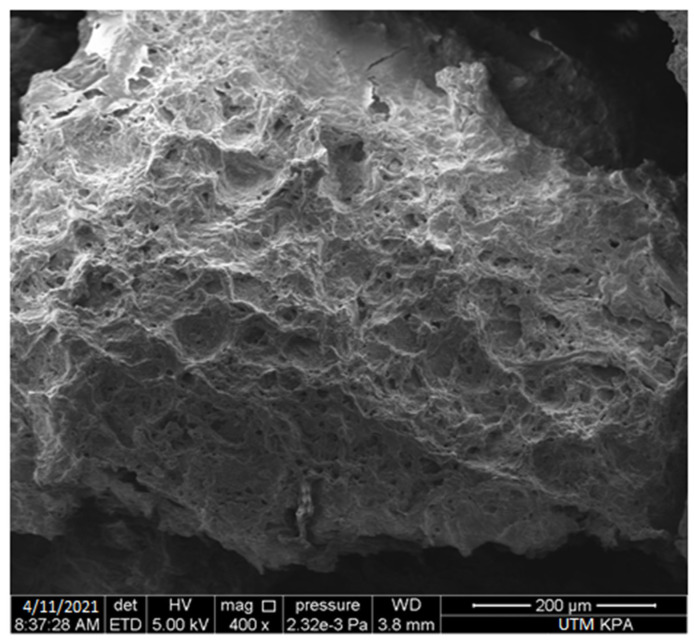
SEM images of F7.

The polymers used in the formulation of tablets results in swelling of the tablets. The result of the scanning electron microscope (SEM) showed that micropores were formed as a result of water absorption (Figure 4).

The formulated tablets exhibited a % swelling for 12 h. The combination of polymers used in the formulation of the tablets exhibited maximum dissolution medium uptake. The tablets formulated with the combination of sodium alginate, chitosan, and HPMC exhibited a maximum amount of medium uptake. The lower side of the tablet comes in contact with the dissolution medium, as opposed to the upper side. After a specific time interval, the formulated tablet sank completely into the medium and allowed both surfaces to have a percent swelling. Similar results with chitosan were also obtained by Naseem et al., 2022. The prepared formulation (F7) containing a combination of polymers (chitosan, sodium alginate, and HPMC, 1:1:1) exhibited a maximum amount of swelling and water uptake; the reason might be that all the polymers are hydrophilic in nature and absorb the maximum amount of water, resulting in attaining the maximum amount of the percent swelling followed by erosion [24].

### 2.6. In Vitro Release of Drug

**Figure 5:**
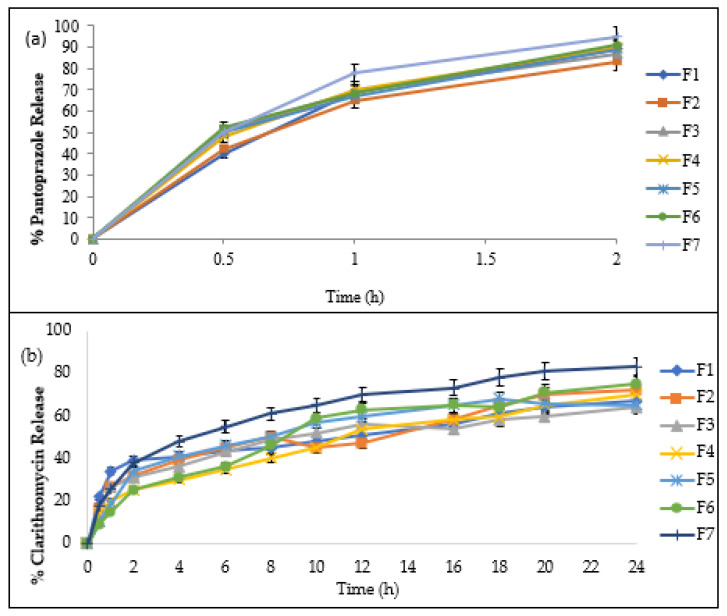
The release profile of (**a**) pantoprazole and (**b**) clarithromycin.

The Type-I USP dissolution apparatus was used for the evaluation of the pantoprazole and clarithromycin drug release profiles. The study was conducted for a time period of 24 h, and the formulated floating tablets exhibited a controlled-release behavior. The study found that pantoprazole was released up to 95% in 2 h. This was due to the use of super disintegrant Primojel^®^. The release of clarithromycin was observed at 83% in 24 h. The immediate release of clarithromycin in the gastric medium demonstrated the rapid degradation of the drug and is not suitable for the gastric treatment of *H. pylori*. Therefore, a combination of polymers was used in the clarithromycin layer, which resulted in drug release over 24 h. The data obtained from this study showed a significant difference in the release of the drug at 24 h (Figure 5a,b) (ANOVA; *p* < 0.05). The difference in the % release of different formulations was observed between 2 and 10 h. This was due to the difference in the % swelling of the floating tablets during this time. The control rate of the drug release from the floating tablets was achieved with the addition of suitable polymers, i.e., HPMC, chitosan, and alginate. Chitosan, when used alone, resulted in an initial burst release of clarithromycin; this is attributed to the partially protonated amine groups of chitosan in the dissolution medium (pH 1.2–2.2). The electrostatic repulsion of the cationic group and high matrix swelling allowed the uptake in the dissolution medium. Chitosan, when combined with alginate and coming in contact with the dissolution medium, swelled and formed a gel layer. The release of the drug was controlled by this gel layer, which acted as a barrier to the drug release. The drug has to pass through this gel layer (barrier) before entering the dissolution medium. As the process of dissolution continues, the thickness of the gel layer increases, which results in a decrease and more controlled drug release. These results are in accordance with the previous studies conducted by Thapa and Jeong, 2018 [25]. The hydration property of sodium alginate is responsible for modifying the drug release from the floating tablets. Sodium alginate at a pH below three is converted to alginic acid, which has the ability to swell upon hydration, and the drug release depends on diffusion through the polymer matrix. These factors control the release of drugs and also influence the release mechanism [26]. The study showed that a higher diffusion path length and swelling contributed to a lower cumulative percentage release of the drug.

### 2.7. Release Kinetics of Drug

The obtained data were subjected to the different kinetic models to evaluate the release pattern of the drug (Table 4). The data were fit to different kinetic models. The power law equation was better suited to our data. The value of “n’ obtained from the formulated transdermal patches ranged between 0.49 and 0.61. The value of “*n*” for the formulated tablets showed that most of the formulations exhibited anomalous non-Fickian transport, whereas F3 and F6 exhibited quasi-Fickian diffusion. The value of *n* suggests that the rate of water uptake into the tablet and the drug release was controlled by diffusion through the gel layer and the swelling/erosion process.

### 2.8. Stability Studies

Stability is one of the important parameters for pharmaceutical dosage forms. Stability studies were performed for the formulation F7 containing chitosan, alginate, and HPMC in a ratio of 1:1:1 at 40 °C for three months. The results of the stability study are shown in Table 5. It was found that no significant difference in drug content, floating behavior, and in vitro release was observed at Month 0 and after three months.

## 3. Conclusions

A bilayer gastro-retentive tablet of pantoprazole sodium (instant layer) and clarithromycin HCl (controlled layer) was prepared successfully. Primojel was used in the instant layer, and a combination of polymers was used for the controlled layer formulation. The formulation F7 containing chitosan, sodium alginate, and HPMC polymers (1:1:1) exhibited a maximum amount of percent swelling (87%) as compared to other formulations. Pantoprazole was released up to 95% in 2 h, whereas clarithromycin exhibited 87% drug release from the controlled layer of the formulated tablets in a time period of 24 h. The use of polymers results in the formation of micropores in the matrix, which controls the release of drugs. The present study concluded that the use of chitosan, sodium alginate, and HPMC in the formulation of bilayer floating tablets has the ability to produce controlled drug delivery for 24 h in the stomach. Moreover, these formulations offer the combination of two drugs in a single tablet formulation and can be the best treatment option for local infections such as gastric ulcers. The current study is limited to in vitro characterization, swelling, floating, and release behavior. In vivo studies using animal models are recommended for future studies.

## 4. Materials and Methods

### 4.1. Materials

Clarithromycin HCl and pantoprazole sodium (Sigma-Aldrich, Chemie, Germany) were the model drugs used in this study. HPMC-K-15M, PVP-K30, chitosan, sodium alginate, sodium bicarbonate (NaHCO_3_), and sodium starch glycolate (primojel) (Dow Chemical, Michigan, United States) and Avecil (Microcrystalline cellulose) PH˗102, magnesium stearate, talc powder, and HCl (Sigma Aldrich, Germany) were used for the formulation of the bilayer floating tablets. Distilled water was prepared in ADDL using a distillation apparatus. All the chemicals used in the study were of analytical grade.

### 4.2. Formulation of Gastro Floating Bilayer Matrix Tablets

#### 4.2.1. Preparation of Immediate-Release Layer of Pantoprazole Sodium

A super disintegrant (Primojel^®^) was used to prepare rapidly dissolving pantoprazole tablets. The direct compression method was used to prepare 100 mg tablets (Table 6). The ingredients were passed through a 40 mesh to remove the large granules. The ingredients (drug and other excipients) were mixed geometrically in a clean and dry environment. Magnesium stearate and talc were added, appropriately mixed using a polyethene bag, and analyzed for pre-compression and post-compression tests. A single-punch tableting machine (Erweka, Germany) with a flat round punch (15 mm diameter) was used for the compression of the pantoprazole sodium formulations (Table 6) [27].

#### 4.2.2. Sustained-Release Layer of Clarithromycin

The drug was mixed with varying amounts of inactive ingredients of sodium alginate, chitosan, and HPMC K15 (F1 CR–F7 CR) and passed through a sieve with a 40-mesh screen to obtain the ingredients uniformly. The physicochemical properties of the formulations were improved with the addition of talcum, magnesium stearate, NaHCO_3_, avicel PH 102, and PVP K30 (Table 7). PE was used to mix the ingredients for 5 min and passed through a sieve of 40 mesh. A single-punch machine with a flat round punch (15 mm diameter) was used to compress the formulations [28].

### 4.3. Preparation of Bilayer Tablets

The pre-formulated mixed powder of a fast-release layer of pantoprazole was compressed in a single-punch tableting machine (Erweka, Germany). The weight of the prepared layer was measured at 100 mg, and the pressure of the machine was maintained slightly lower as compared to the actual pressure. The tablets of the pantoprazole sodium instant layer were prepared separately, and the same procedure was used for the preparation of the clarithromycin layer of the controlled-release tablets (500 mg). These layers were compressed on top of each other as bilayer tablets. The compression parameters were modified to the desirable limit to withstand the appropriate hardness of the tablets, and the tablets were named as F1–F7, containing both the instant layer of pantoperazole and the controlled layer of clarithromycin, and were evaluated for further studies [29].

### 4.4. Pre-Compression Studies

#### 4.4.1. Angle of Repose

The friction or resistance to the movements of particles can be predicted using the angle of repose by the funnel technique. The lower end of the funnel was placed over the heap of powder. From the top of the funnel, a specified weighed amount of powder was poured freely and slowly. A cone-like structure was formed on the surface. The cone-like structure’s diameter was calculated [30].

#### 4.4.2. Bulk Density

When the powder is poured into a container, the bulk density is defined as the ratio of the known mass to the bulk volume covered by the powder. First, the powder was weighed accurately using an analytical balance, and then, it was poured into a graduated cylinder. The bulk volume was recorded, and the bulk density was computed (Equations (5) and (6)) [31].

Bulk density = bulk mass / bulk volume (1)

ρ = m/V(2)

#### 4.4.3. Tapped Density

The graded cylinder was mechanically tapped to obtain the final capacity in order to quantify the tapped volume. The value for the tapped density was determined by the mass of the specified powder to the final tapped volume. The tapped density was determined by placing the weighed amount of powder in the graduated cylinder and, then, tapping until it reached the minimum value, then this process was repeated three times, and the reading was recorded and calculated as the mean ± SD (Equations (7) and (8)) [32].

Tapped density = powder mass/tapped volume (3)

ρ_(t)_ = m/V_(t)_ (4)

#### 4.4.4. Carr’s Compressibility Index

The propensity of the powder was calculated with the data of Carr’s compressibility index. This technique is also referred to as Carr’s index and is often utilized for the evaluation of the flow behavior of powder. The compressibility index was calculated using Equation (3) [33].

Compressibility index = density(tapped) − density(bulk)/density(tapped) × 100(5)

#### 4.4.5. Hausner’s Ratio

Hausner’s ratio is related to the compressibility index. Hausner’s ratio is also used for the evaluation of the flow property of a powder. Hausner’s ratio is non-measurable and can be calculated using Equation (4) [34].

Hausner’s ratio = density(tapped)/density(bulk)(6)

### 4.5. Post-Compression Study

#### 4.5.1. Hardness Test

Appropriate hardness is necessary to tolerate mechanical forces during the processing, handling, transportation, and storage of tablets. The hardness of each formulation was tested using a hardness tester (Erweka TB 24 Apparatus, Langen, Germany). For each batch, a total of 5 tablets were randomly sampled for hardness. The results obtained were calculated as the mean ± SD.

#### 4.5.2. Weight Variation

An analytical balance (M Series, BEL Engineering, Monza, Italy) was used for the weight variation of the formulated tablets. Randomly, ten tablets were chosen, and each was weighed separately. The individual weights of the tablets were taken, and the average weights of the tablets were calculated. The results are presented as the mean ± SD [35].

#### 4.5.3. Tablets’ Dimensions

A vernier caliper (Insize, Digital) was used to evaluate the dimensions of the formulated tablets. Randomly, five tablets were selected and individually measured in the assessment of the tablets’ dimensions (thickness and diameter). The results collected were averaged as the mean ± SD [35].

#### 4.5.4. Friability

The drum of a friability testing device (Erweka TA3R, Germany) was filled with a sample of 10 tablets following the USP specifications, and the apparatus was run for 4 min at 100 rpm. The tablets were weighed once more, and the amount of weight reduction was calculated based on Equation (9). The test was repeated three times, with the findings represented as the mean ± SD [8].

% Friability = (W1 − W2)/W1 × 100(7)

#### 4.5.5. Floating Lag Time and Floating Time

The amount of time it takes for a tablet to begin floating after being dissolved in stomach fluid is known as the floating lag time [36]. A tablet was placed in a beaker containing 900 mL of 0.1N HCl to measure the floating lag time, and the amount of time it took the tablet to reach the medium’s surface was timed. Six tablets had their floating lag time evaluated independently. The mean and standard deviation of the floating lag time were calculated [37]. Floating time refers to how long tablets stay afloat in the medium. A tablet was placed in 900 mL of 0.1N HCl in the vessel of a USP dissolving Type II apparatus to evaluate the floating duration. The vessel was operated at 100 rpm at 37 °C to replicate the stomach environment. The time was measured for six tablets used in this experiment by a stopwatch. The data’s mean and standard deviation were calculated. [38].

### 4.6. Characterization of Drug and Excipients

This study was carried out to evaluate the compatibilities of the drug–excipient used in the formulation of the tablets.

#### 4.6.1. Attenuated Total Reflectance/Fourier Transform Infrared Spectroscopy

The attenuated total reflectance/Fourier transform infrared spectroscopy (ATR-FTIR) spectra of the drug and excipients were individually recorded and compared with the spectra of the formulations. The formulated tablets were crushed, and the powder was subjected to ATR-FTIR spectrophotometry (Perkin Elmer, Waltham, MA, USA) [32]. The sample was placed on zinc selenide and scanned directly without further processing. The FTIR spectra were scanned between 4000 and 500 cm^−1^. Three ranges were taken for each sample, and the results were averaged.

#### 4.6.2. Differential Scanning Calorimetry Analysis

The Perkin Elmer JADE DSC equipment was used to obtain DSC thermograms for the medication and formulation (F7). A sample of pure substances (3–5 mg) was weighed, sealed in a typical aluminum pan, and then, put on the apparatus for analysis. At a scanning rate of 10 °C/min from 0 to 300 °C, the sample’s thermal behavior was examined. The outcomes were averaged after each sample, conducted in triplicate.

### 4.7. Drug Content Uniformity

#### 4.7.1. Drug Content of Clarithromycin

The drug content uniformity of clarithromycin was analyzed by the HPLC method. The chromatographic system used for the investigation was a Shimadzu (Kyoto, Japan), equipped with a UV–visible detector.

##### Procedure for Evaluating Drug Content

In a beaker, 100 mL of distilled water was taken, and 4.5 g of KH_2_PO_4_ was added. The mobile phase was comprised of methanol: buffer (65:35), and the pH was maintained at 4.0 using ortho-phosphoric acid. A C18 column (4.6 mm × 250 mm, 5µ) was utilized. The flow rate was set at 1 mL/min for 15 minutes with the injection volume adjusted to 20 L. The wavelength was adjusted at 210 nm. The retention time of clarithromycin was 4.1 min. A volumetric flask was filled with powder containing 100 mg of clarithromycin after a total of six tablets from each formulation were crushed with a pestle and mortar. Then, methanol was added to a volumetric flask, sonicated for 2 min, and the final volume attained with the addition of methanol. The known concentration of 1 mg/mL was de-gassed and filtered. The filtrate (5 mL) was incorporated into a 50 mL volumetric flask, and the mobile phase was introduced to make the final volume and concentration of 0.1 mg/mL. From each formulation, a 20 µL volume was injected into the chromatogram individually. The major peaks of the chromatogram were selected and measured, and the percentage of clarithromycin concentration was calculated (Equation (10)) [38]. This method was validated and used for the quantification of clarithromycin in pure, as well as matrix floating bilayer tablet form.

% Drug = sample peak area / standard peak area × 100 (8)

#### 4.7.2. Drug Content of Pantoprazole

UV–visible spectrophotometry at a wavelength of 286 nm was used to assess the uniformity of the drug content of pantoprazole. Randomly, six tablets were selected from the formulation and crushed in a pestle and mortar. The crushed powder (600 mg) comprising 40 mg of pantoprazole was put in a 0.1 N HCl solution (pH 1.2) in a 10 mL volumetric flask. The volumetric flask was sonicated for 2 min to achieve efficient dissolution. Then, the drug solution (1 mL) was diluted with 0.1 N HCl to make the concentration at 0.016 mg/mL. The same technique was employed for the preparation of standard drug solutions (Equation (11)) [39]. The method used for quantifying pantoprazole was validated and used for the pure drug from floating bilayer tablets.

% Drug content = sample absorbance/standard abs × 100(9)

### 4.8. Swelling and Erosion Studies

Three tablets were weighed and added to the dissolution media. The USP dissolve equipment II (Erweka GmbH, Langen, Germany) was used to measure the absorption and swelling percentages of the tablets under the same circumstances as the drug release study described in Section 4.10. At various times, the tablets were gently removed from the medium (0.5, 1, 2, 4, 6, 8, 12, and 24 h). The excess liquid from the tablet surfaces was wiped off using filter paper and, then, weighed and dried in a drying oven at 60 °C until reaching a constant dry weight. Equations (12) and (13) were used to the compute water intake and swelling rate:

(10) $$\%\;\mathrm{water}\;\mathrm{uptake}=\frac{\left(\mathrm{Ww}-\mathrm{Wi}\right)}{\mathrm{Wi}}*100$$

(11) $$\%\;\mathrm{swelling}\;\mathrm{index}=\frac{\left(\mathrm{Wi}-\mathrm{Wd}\right)}{\mathrm{Wi}}*100$$

where Wi represents the initial weight of the tablet, Ww its wet weight, and Wd its dry weight; mean values with the standard deviation are displayed.

### 4.9. SEM Analysis

A floating tablet at 12 h was taken, dried, and subjected to scanning electron microscopy for surface morphological analysis (Carl Zeiss, GmbH, 73447 Oberkochen, Germany). The dried tablet was fixed with a carbon stub. The prepared studs were analyzed directly under a scanning electron microscope. Images of the specified portion were taken.

### 4.10. In Vitro Release of Drug

The Dissolution USP Apparatus I (basket method) was used to assess the drug release using 900 mL of a 0.1 N HCl solution (pH 1.2) as the dissolution media for the sink at 37 ± 0.5 °C and 100 rpm. At predetermined intervals, aliquots (5 mL) were collected, filtered through 0.45 m filters (Nylon, Whatman, Maidstone, UK), and an equal volume of fresh solvent replaced. As previously mentioned, clarithromycin and pantoprazole were quantified using HPLC and UV spectroscopy. The results are then given as the mean ± SD.

### 4.11. Release Kinetics

#### 4.11.1. Zero-Order Kinetics

The zero-order kinetic equation or model was carried out to investigate the kinetic profiling of the prepared formulations (F1–F7). The zero-order kinetics was carried out using the following equation:

W = k1 × t (12)

where W represents drug release, k1 represents the zero-order-kinetic constant, and t represents the time for drug release.

#### 4.11.2. First-Order Kinetics

The first-order kinetics equations or model was first proposed by Gibaldi and Feldman (1967) and later by Wagner (1969). The first-order kinetics was carried out using the following equation:

ln (100 − W) = ln 100 − k2t(13)

where W represents the release of the drug, k2 represents the first-order kinetics constant, and t represents the time for drug release.

#### 4.11.3. Hixon–Crowell Model

The Hixon–Crowell model was used for the first time by Hixon–Crowell in 1931. This method was carried out for evaluating the kinetic profiling of the prepared formulations (F1–F7). The Hixon–Crowell model was carried out using the following equation:

(100 − W) 1/3 = 100 (1/3) − k3t(14)

where W represents the release of the drug with respect to time, k3t represents the relationship of the surface volume to Hixon–Crowell, and t represents the release of the drug with respect to time.

#### 4.11.4. Higuchi Model

This method was first used by Higuchi. The Higuchi method was used for the evaluation of the prepared formulations (F1–F7). The Higuchi model was carried out using the following equation:

W = k4t × 1/2(15)

where W represents the release of the drug, k4 represents the Higuchi rate of dissolution constant, and t represents the release of the drug.

#### 4.11.5. Korsmeyer–Peppas model 

The optimized bilayer tablets were selected for release kinetics using non-linear regression to fit the dissolution data into the Korsmeyer–Peppas model (Equation (14)) [36]:

Mt/M∞ = Kt^n^ (16)

where Mt/M∞ is the cumulative fraction (percentage) of the medication released at time t, k denotes the release constant, and *n* denotes the release exponent.

*n* = 0.5 denotes mere Fickian diffusion release; *n* = 1.0 denotes just relaxation (Case II transport); 0.5 *n* 1.0 denotes anomalous transport controlled by both mechanisms; *n*-values greater than 1.0 denote Super Case II transport. Mt/M = k0 t is a specific instance of the Korsmeyer–Peppas model with *n* = 1.0, where k0 indicates the zero-order release constant [40].

### 4.12. Stability Studies

A stability study for the formulation (F7) was performed according to the ICH recommendations at 40 °C/75 ± 5% RH using a stability chamber (Thermolab TH 90s, Maharashtra, India) for three months. The drug content, floating behavior, and in vitro drug release profile of the prepared tablets were evaluated.

### 4.13. Statistical Analysis

Triplicate results were gathered, computed, and averaged (mean SD) in this investigation. IBM’s SPSS Version 16 software (Chicago, IL, USA) was used for the statistical analysis. The significance level was set at *p* = 0.05. The statistical method for the study was one-way ANOVA/post hoc analysis along with Tukey’s honestly significant difference test.

## Figures and Tables

**Table 1:** Instant-release layer (F1 IR-F3 IR) of pantoprazole and sustained-release layer (F1 FL-F7 FL) of clarithromycin flow characteristics.

F. Layer-IR	Bulk Density (mg/cm^3^)	Tapped Density (mg/cm^3^)	Compressibility Index (%)	Hausner’s Ratio	Angle of Repose (θ)
F1 IR	0.862 ± 0.021	0.940 ± 0.036	8.298 ± 0.0122	1.09 ± 0.031	29.47
F2 IR	0.868 ± 0.007	0.937 ± 0.028	7.364 ± 0.0115	1.08 ± 0.034	29.62
F3 IR	0.871 ± 0.030	0.936 ± 0.023	6.944 ± 0.025	1.075 ± 0.078	29.70
F1 FL	0.797 ± 0.023	0.921 ± 0.005	13.46 ± 0.018	1.156 ± 0.012	29.71
F2 FL	0.783 ± 0.082	0.919 ± 0.014	14.80 ± 0.0115	1.174 ± 0.043	29.42
F3 FL	0.819 ± 0.029	0.899 ± 0.070	8.899 ± 0.0173	1.098 ± 0.056	30.14
F4 FL	0.833 ± 0.018	0.961 ± 0.037	13.32 ± 0.0015	1.154 ± 0.041	30.53
F5 FL	0.784 ± 0.031	0.865 ± 0.009	9.364 ± 0.052	1.103 ± 0.032	30.02
F6 FL	0.850 ± 0.023	0.941 ± 0.011	9.671 ± 0.001	1.107 ± 0.019	30.31
F7 FL	0.847 ± 0.016	0.925 ± 0.081	8.432 ± 0.026	1.092 ± 0.063	30.13

Data are shown as the mean ± the standard deviation; *n* = 3.

**Table 2:** Post-compression tests of the formulated tablets.

F. Code	Thickness (mm)	Hardness (N/cm^2^)	Weight Uniformity (mg)	Friability (%)	Drug Content (%)	
					Clarithromycin	Pantoprazole
F1	2.75 ± 0.1	68 ± 1.2	599.1 ± 2.4	0.73 ± 0.02	98.2 ± 1.3	100.5 ± 1.5
F2	2.68 ± 0.3	69 ± 0.4	601.7 ± 3.1	0.79 ± 0.05	99.1 ± 1.1	97.9 ± 1.4
F3	2.65 ± 0.1	68 ± 1.1	598.3 ± 3.2	0.64 ± 0.04	98.7 ± 1.4	99.3 ± 1.7
F4	2.73 ± 0.2	73 ± 0.9	604.1 ± 1.6	0.58 ± 0.09	100.1 ± 1.6	98.1 ± 1.3
F5	2.69 ± 0.3	75 ± 1.3	602.8 ± 1.9	0.79 ± 0.05	97.9 ± 1.8	99.8 ± 1.2
F6	2.70 ± 0.4	71 ± 1.2	600.5 ± 1.1	0.72 ± 0.04	99.6 ± 1.5	98.5 ± 1.6
F7	2.68 ± 0.3	72 ± 1.2	602.3 ± 2.0	0.63 ± 0.07	100.7 ± 1.1	97.4 ± 1.8

Data are shown as the mean ± the standard deviation; *n* = 3.

**Table 3:** The floating characteristics of the formulated tablets.

F. Codes	Floating Lag Time (min:s)	Total Floating Duration (h)
F1	0:24 ± 0.17	18
F2	0:16 ± 0.13	17
F3	0:43 ± 0.10	18
F4	0:12 ± 0.06	18
F5	0:39 ± 0.15	22
F6	1:38 ± 0.14	23
F7	1:01 ± 0.14	24

Data are shown as the mean ± the standard deviation; *n* = 3.

**Table 4:** Values for the release exponent and linearity in kinetic models (Korsmeyer–Peppas kinetics).

F. Code	Zero-Order		1st-Order		Higuchi		Hixon–Crowell		Korsmeyer–Peppas		
	K ± SD	R^2^	K ± SD	R^2^	K ± SD	R^2^	K ± SD	R^2^	K ± SD	R^2^	*n*
F1	1.13 ± 0.113	0.976	1.32 ± 0.132	0.534	2.29 ± 0.132	0.954	1.54 ± 0.134	0.992	0.143 ± 0.034	0.975	0.61
F2	1.32 ± 0.119	0.987	1.13 ± 0.167	0.643	1.43 ± 0.243	0.954	1.34 ± 0.232	0.978	0.162 ± 0.332	0.981	0.61
F3	1.14 ± 0.142	0.967	1.19 ± 0.132	0.623	1.54 ± 0.423	0.976	1.65 ± 0.154	0.989	0.045 ± 0.343	0.970	0.49
F4	1.17 ± 0.114	0.976	1.32 ± 0.176	0.554	1.24 ± 1.254	0.945	1.23 ± 0.234	0.978	0.065 ± 0.123	0.984	0.515
F5	1.28 ± 0.128	0.979	1.28 ± 0.121	0.525	2.32 ± 0.034	0.976	1.54 ± 0.245	0.986	0.056± 0.045	0.981	0.535
F6	1.26 ± 0.126	0.987	1.19 ± 0.134	0.643	1.54 ± 0.132	0.923	1.25 ± 0.254	0.987	0.112 ± 0.234	0.980	0.49
F7	1.18 ± 0.229	0.983	1.32 ± 0.122	0.648	1.13 ± 0.124	0.987	1.54 ± 0.165	0.990	0.054 ± 0.316	0.982	0.553

**Table 5:** Stability studies of formulation F7.

Time (Month)	Evaluation Parameters					
	Drug Content		FLT (s)	TFT (h)	% Drug Release	
	Cln	Ptz			Cln	Ptz
0	100.7 ± 1.1	97.4 ± 1.8	60	>24	99.4	97.3
1	99.5 ± 1.3	97.1 ± 1.4	58	>24	99.3	96.5
2	99.3 ± 0.9	96.6 ± 1.1	55	>24	98.7	95.2
3	99.1 ± 1.5	96.2 ± 2.1	59	>24	99.1	96.4

Note: clarithromycin (Cln), pantoprazole (Ptz), floating lag time (FLT), and total floating time (TFT).

**Table 6:** Ingredients of the immediate-release layer of pantoprazole sodium.

Ingredients	F1 IR (mg)	F2 IR (mg)	F3 IR (mg)
Pantoprazole sodium	40	40	40
Sodium starch glycolate (Primojel)	05	10	15
Avicel 102	44	39	34
Magnesium stearate	05	05	05
Talcum powder	06	06	06
Total weight	100	100	100

**Table 7:** Ingredients of the slow-release floating layer of clarithromycin.

Ingredients (mg)	F1 CR	F2 CR	F3 CR	F4 CR	F5 CR	F6 CR	F7 CR
Clarithromycin HCl	250	250	250	250	250	250	250
Chitosan	150	---	---	75	---	75	50
HPMC K15M	---	150	---	75	75	---	50
Sodium alginate	---	---	150	---	75	75	50
NaHCO_3_	30	30	30	30	30	30	30
PVP K30	20	20	20	20	20	20	20
Talcum powder	10	10	10	10	10	10	10
Magnesium stearate	10	10	10	10	10	10	10
Avicel 102	30	30	30	30	30	30	30
Weight/tablet	500	500	500	500	500	500	500

## Data Availability

Not applicable.

## References

[1] Patil T., Pawar A., Korake S., Patil R., Pawar A., Kamble R. (2022). Green synthesis of polyacrylamide grafted Neem Gum for gastro retentive floating drug delivery of Ciprofloxacin Hydrochloride: In vitro and in vivo evaluation. J. Drug Deliv. Sci. Technol..

[2] Karole A., Parvez S., Thakur R.S., Mudavath S.L. (2022). Effervescent based nano-gas carrier enhanced the bioavailability of poorly aqueous soluble drug: A comprehensive mechanistic understanding. J. Drug Deliv. Sci. Technol..

[3] Tripathi J., Thapa P., Maharjan R., Jeong S.H. (2019). Current State and Future Perspectives on Gastroretentive Drug Delivery Systems. Pharmaceutics.

[4] Vrettos N.-N., Roberts C.J., Zhu Z. (2021). Gastroretentive Technologies in Tandem with Controlled-Release Strategies: A Potent Answer to Oral Drug Bioavailability and Patient Compliance Implications. Pharmaceutics.

[5] Kotreka U.K., Adeyeye M.C. (2011). Gastroretentive floating drug-delivery systems: A critical review. Crit. Rev. Ther. Drug Carr. Syst..

[6] Pawar V.K., Kansal S., Garg G., Awasthi R., Singodia D., Kulkarni G.T. (2011). Gastroretentive dosage forms: A review with special emphasis on floating drug delivery systems. Drug Deliv..

[7] Jin G., Ngo H.V., Wang J., Cui J.-H., Cao Q.-R., Park C., Jung M., Lee B.-J. (2022). Design and evaluation of in vivo bioavailability in beagle dogs of bilayer tablet consisting of immediate release nanosuspension and sustained release layers of rebamipide. Int. J. Pharm..

[8] Maddiboyina B., Hanumanaik M., Nakkala R.K., Jhawat V., Rawat P., Alam A., Foudah A.I., Alrobaian M.M., Shukla R., Singh S. (2020). Formulation and evaluation of gastro-retentive floating bilayer tablet for the treatment of hypertension. Heliyon.

[9] Efentakis M., Naseef H., Vlachou M. (2010). Two- and three-layer tablet drug delivery systems for oral sustained release of soluble and poorly soluble drugs. Drug Dev. Ind. Pharm..

[10] Parsa H., Zangivand A.A., Hajimaghsoudi L. (2012). The effect of pentoxifylline on chronic venous ulcers. Wounds Compend. Clin. Res. Pract..

[11] Cui G., Yuan A., Li Z. (2022). Occurrences and phenotypes of RIPK3-positive gastric cells in Helicobacter pylori infected gastritis and atrophic lesions. Dig. Liver Dis..

[12] Valladales-Restrepo L.F., Correa-Sánchez Y., Aristizábal-Carmona B.S., Machado-Alba J.E. (2022). Treatment regimens used in the management of Helicobacter pylori in Colombia. Braz. J. Infect. Dis..

[13] Hussein R.A., Al-Ouqaili M.T.S., Majeed Y.H. (2021). Detection of clarithromycin resistance and 23SrRNA point mutations in clinical isolates of Helicobacter pylori isolates: Phenotypic and molecular methods. Saudi J. Biol. Sci..

[14] Rodvold K.A. (1999). Clinical Pharmacokinetics of Clarithromycin. Clin. Pharmacokinet..

[15] Jain N., Jain P., Parmar T., Parkhe G., Jain S.K. (2016). Preparation and Evaluation of Bi-Layer Tablets of Pantorazole and Clarithromycin. J. Pharm. Educ. Res..

[16] Peppas N.A. (1985). Analysis of Fickian and non-Fickian drug release from polymers. Pharm. Acta Helv..

[17] Solunke R.S., Khade G.A., Krishnmurthy, Deshmukh M.T., Shete R.V., Kore K.J. (2020). Development and Evaluation of Pantoprazole Sodium Floating Gel. Res. J. Pharm. Technol..

[18] Guarnizo-Herrero V., Torrado-Salmerón C., Pabón N.S.T., Durán G.T., Morales J., Torrado-Santiago S. (2021). Study of Different Chitosan/Sodium Carboxymethyl Cellulose Proportions in the Development of Polyelectrolyte Complexes for the Sustained Release of Clarithromycin from Matrix Tablets. Polymers.

[19] Prakash G., Chandra S.A., Sandhya P., Bidur C., Samir D. (2020). Pharmacopoeial comparison of in-process and finished product quality control test for pharmaceutical tablets. GSC Biol. Pharm. Sci..

[20] British Pharmacopoeia (2009). Her Majesty’s Stationery Office.

[21] Patel S.S., Patel N.M. (2009). Flowability Testing of Directly Compressible Excipients According to British Pharmacopoeia. J. Pharm. Res..

[22] Kumar V.S., Rijo J., Sabitha M. (2018). Guargum and Eudragit^®^ coated curcumin liquid solid tablets for colon specific drug delivery. Int. J. Biol. Macromol..

[23] Sharma D., Dev D., Prasad D.N., Hans M. (2019). Sustained release drug delivery system with the role of natural polymers: A review. J. Drug Deliv. Ther..

[24] Naseem F., Shah S.U., Rashid S.A., Farid A., Almehmadi M., Alghamdi S. (2022). Metronidazole Based Floating Bioadhesive Drug Delivery System for Potential Eradication of H. pylori: Preparation and In Vitro Characterization. Polymers.

[25] Thapa P., Jeong S.H. (2018). Effects of Formulation and Process Variables on Gastroretentive Floating Tablets with A High-Dose Soluble Drug and Experimental Design Approach. Pharmaceutics.

[26] Sharma V., Devi J. (2021). Design of Cellulose Derivative and Alginate Based Smart Polymers to Develop Stomach Specific Floating Drug Delivery System. Curr. Smart Mater..

[27] Mittal S., Pawar S. (2018). Design and evaluation of buccal mucoadhesive tablets of pantoprazole sodium. Eur. J. Pharm. Med. Res..

[28] Rajeswari S., Kudamala S., Murthy K.V.R. (2017). Development, Formulation and Evaluation of A Bilayer Gastric Retentive Floating Tablets of Ranitidine Hcl and Clarithromycin using Natural Polymers. Int. J. Pharm. Pharm. Sci..

[29] Alam M., Shah K.U., Khan K.A., Nawaz A., Bibi H., Razaque G., Niazi Z.R., Alfatama M. (2021). Formulation and in vitro Evaluation of Effervescent Bilayer Floating Controlled Release Tablets of Clarithromycin and Famotidine. Res. J. Pharm. Technol..

[30] Müller D., Fimbinger E., Brand C. (2021). Algorithm for the determination of the angle of repose in bulk material analysis. Powder Technol..

[31] Kamal M.M., Salawi A., Lam M., Nokhodchi A., Abu-Fayyad A., El Sayed K.A., Nazzal S. (2020). Development and characterization of curcumin-loaded solid self-emulsifying drug delivery system (SEDDS) by spray drying using Soluplus^®^ as solid carrier. Powder Technol..

[32] Chaudhari K.D., Nimbalwar M.G., Singhal N.S., Panchale W.A., Manwar J.V., Bakal R.L. (2021). Comprehensive review on characterizations and application of gastro-retentive floating drug delivery system. GSC Adv. Res. Rev..

[33] Özalp Y., Onayo M.M., Nailla J. (2020). Evaluation of lactose-based direct tabletting agents’ compressibility behaviour using a compaction simulator. Turk. J. Pharm. Sci..

[34] Venkatesh D.N., Meyyanathan S., Shanmugam R., Zielinska A., Campos J., Ferreira J., Souto E. (2019). Development, in vitro release and in vivo bioavailability of sustained release nateglinide tablets. J. Drug Deliv. Sci. Technol..

[35] Thakur S., Ramya K., Shah D.K., Raj K. (2021). Floating Drug Delivery System. J. Drug Deliv. Ther..

[36] Rao G.K., Mandapalli P.K., Manthri R., Reddy V.P. (2013). Development and in vivo evaluation of gastroretentive delivery systems for cefuroxime axetil. Saudi Pharm. J..

[37] Raza A., Bukhari N.I., Karim S., Hafiz M.A., Hayat U. (2017). Floating tablets of minocycline hydrochloride: Formulation, in-vitro evaluation and optimization. Future J. Pharm. Sci..

[38] Yin L., Qin C., Chen K., Zhu C., Cao H., Zhou J., He W., Zhang Q. (2013). Gastro-floating tablets of cephalexin: Preparation and in vitro/in vivo evaluation. Int. J. Pharm..

[39] Sharaf M., Arif M., Khan S., Abdalla M., Shabana S., Chi Z., Liu C. (2021). Co-delivery of hesperidin and clarithromycin in a nanostructured lipid carrier for the eradication of Helicobacter pylori In vitro. Bioorganic Chem..

[40] Latha S., Selvamani P., Suganya G., Raj D.B.T.G., Pal T.K. (2021). Preparation and In-vitro Evaluation of Pantoprazole Sodium Magnetic Microspheres by Emulsion Solvent Evaporation Method. BioNanoScience.

